# Dual-Layered Nanogel-Coated Hollow Lipid/Polypeptide Conjugate Assemblies for Potential pH-Triggered Intracellular Drug Release

**DOI:** 10.1371/journal.pone.0092268

**Published:** 2014-03-20

**Authors:** Wen-Hsuan Chiang, Wen-Chia Huang, Ming-Yin Shen, Che-Hsu Wang, Yi-Fong Huang, Sung-Chyr Lin, Chorng-Shyan Chern, Hsin-Cheng Chiu

**Affiliations:** 1 Department of Biomedical Engineering and Environmental Sciences, National Tsing Hua University, Hsinchu, Taiwan; 2 Department of Surgery, National Taiwan University Hospital-Hsinchu Branch, Hsinchu, Taiwan; 3 Department of Chemical Engineering, National Chung Hsing University, Taichung, Taiwan; 4 Department of Chemical Engineering, National Taiwan University of Science and Technology, Taipei, Taiwan; The Ohio State University, United States of America

## Abstract

To achieve effective intracellular anticancer drug delivery, the polymeric vesicles supplemented with the pH-responsive outlayered gels as a delivery system of doxorubicin (DOX) were developed from self-assembly of the lipid/polypeptide adduct, distearin grafted poly(γ-glutamic acid) (poly(γ-GA)), followed by sequential deposition of chitosan and poly(γ-GA-co-γ-glutamyl oxysuccinimide)-g-monomethoxy poly(ethylene glycol) in combination with in situ covalent cross-linking on assembly surfaces. The resultant gel-caged polymeric vesicles (GCPVs) showed superior performance in regulating drug release in response to the external pH change. Under typical physiological conditions (pH 7.4 and 37°C) at which the γ-GA/DOX ionic pairings remained mostly undisturbed, the dense outlayered gels of GCPVs significantly reduced the premature leakage of the uncomplexed payload. With the environmental pH being reduced from pH 7.4 to 4.7, the drug liberation was appreciably promoted by the massive disruption of the ionic γ-GA/DOX complexes along with the significant swelling of nanogel layers upon the increased protonation of chitosan chain segments. After being internalized by HeLa cells via endocytosis, GCPVs exhibited cytotoxic effect comparable to free DOX achieved by rapidly releasing the payload in intracellular acidic endosomes and lysosomes. This strongly implies the great promise of such unique GCPVs as an intracellular drug delivery carrier for potential anticancer treatment.

## Introduction

Over the past decades, various nanoassemblies such as liposomes, polymeric micelles and polymeric vesicles (polymersomes) have been exploited extensively as anticancer drug transport vehicles due to their great capability of delivering payloads to tumor regions achieved primarily by the enhanced permeability and retention effects [1], [2], [3], [4], [5], [6]. Although these nanovehicles show promise in selective delivery of therapeutic agents to target sites, several intractable problems (e.g., the premature drug leakage from carriers and poor intracellular drug-release property that lead to an insufficient drug bioavailability for killing cancer cells and undesired side effects) have not been completely overcome yet [7], [8], [9]. In this regard, substantial efforts have been devoted to the development of stimuli-responsive devices as novel drug delivery systems capable of controlling payload release in response to biological stimuli such as temperature [10], [11], pH [12], [13], [14], [15], and redox potential [16], [17], [18]. The stimuli-triggered drug liberation could significantly promote therapeutic efficacy and minimize side effects. Among these stimuli, acidic pH has been frequently adopted as an optimal internal trigger due to the mildly acidic pH existing in tumor tissues and in the intracellular organelles including both endosomes and lysosomes [13], [14]. To achieve the pH-triggered intracellular drug release, various nanovehicles functionalized with pH-responsive structural characteristics have been developed. By contrast, in order to meet the basic requirement of the assembly stability in practical drug delivery application without impairing or even with promoting the stimuli-triggered characteristics, several approaches such as covalent crosslinking [19], [20], [21], mineralization [22], [23], [24], and surface modification [25], [26], [27] of nanoparticles have been supplemented to enhance their structural integrity under varying conditions.

As described by Shuai and co-workers [20], through the formation of disulfide cross-links inside the intermediate layer of polymeric micelles assembled from a triblock copolymer composed of monomethoxy poly(ethylene glycol) (mPEG), 2-mercaptoethylamine-grafted poly(L-aspartic acid) and 2-(diisopropylamino)ethylamine-grafted poly(L-aspartic acid) (PAsp(DIP)) in aqueous solution of pH 10.0, dual pH- and redox-responsive cross-linked micelles were developed as carriers for intracellular delivery of anticancer drug (doxorubicin (DOX)). With being internalized into cancer cells and localized within glutathione-rich acidic lysosomes (ca. pH 5.0), DOX-loaded micelles showed a prompt payload release as a result of both cleavage of the disulfide cross-links of intermediate gel layers and disintegration of PAsp(DIP) cores. Han et al. [23] reported that the hyaluronic acid-based nanoparticles after being mineralized by calcium phosphate exhibited a rather robust structure at pH 7.4 and were utilized as a pH-responsive DOX delivery vehicle. While the solution pH being adjusted from 7.4 to 5.0, the DOX release from the mineralized nanoparticles was significantly promoted by the dissolution of calcium phosphate in weak acidic environment. On the other hand, Nguyen and co-workers [27] developed the polymer-derived nanocages as a potential molecular drug delivery platform by the insertion of the cholesterol-modified poly(acrylic acid) into lipid membranes of liposomes and subsequent covalent crosslinking with alkyne-functionalized diamine linker. These gel-caged liposomes exhibited pH-responsive characteristics capable of triggering the DOX release from the liposomes under mild acidic conditions upon the formation of temporary pores throughout liposomal membranes as a result of the structural variation of gel-like polymer cages.

Distinct from these pioneered studies, a step-by-step polyelectrolyte deposition technique in combination with in situ covalent crosslinking was used in this work to endow polymeric vesicles with highly biocompatible, robust and pH-responsive outlayered gels for improved intracellular DOX delivery efficiency. The proposed approach involves the synthesis of the lipid/polypeptide conjugate, poly(γ-glutamic acid-co-γ-distearin glutamate) (poly(γ-GA-co-γ-DSGA)), followed by the self-assembly of the resultant conjugate into polymeric vesicles in aqueous solution of DOX. Through the sequential deposition of chitosan and poly(γ-GA-co-γ-glutamyl oxysuccinimide)-g-mPEG (poly(γ-GA-co-γ-GAOSu)-g-mPEG) on the outer surfaces of DOX-encapsulated lipid/polypeptide conjugate vesicles upon paired electrostatic attraction and then covalent crosslinking via aminolysis of γ-GAOSu moieties with primary amines of chitosan, drug-loaded gel-caged polymeric vesicles (GCPVs) were thus attained. The physicochemical properties of these unique drug-loaded GCPVs were then characterized in detail. The effects of the dual-layered gels of GCPVs on the in vitro drug release performance were also explored. In addition, the cell uptake of the DOX-loaded GCPVs and their cytotoxicity against human cervical tumor cell line, HeLa cell, were evaluated to demonstrate the GCPVs developed in this study as a rather potential intracellular drug delivery carrier.

## Materials and Methods

### Materials

1,2-Distearoyl-rac-glycerol (distearin) was purchased from Bachem and DOX (in the hydrochloride salt form) obtained from Seedchem. Poly(γ-GA) (M_n_ > 100 kDa) was purchased from Vedan. *N*-Hydroxysuccinimide (NHS) and *N,N’*-dicyclohexylcarbodiimide (DCC) were obtained from Aldrich and Acros, respectively. Chitosan was purchased from Fluka. Synthesis and characterization of mPEG-NH_2_ (5000 g/mol) was described in our previous studies [28], [29]. HeLa cells were purchased from Food Industry Research and Development Institute of Taiwan. Dulbecco’s modified Eagle medium (DMEM) and Hoechst 33258 were purchased from Invitrogen. The reagent 3-(4,5-dimethylthiazol-2yl)-2,5-diphenyltetrazolium bromide) (MTT) was purchased from TCI. Deionized water was produced from Milli-Q Synthesis (18 MΩ, Millipore). All other chemicals were reagent grade and used as received.

### Fractionation of Poly(γ-GA)

Poly(γ-GA) (1.0 g) was dissolved in aqueous solution of NaHCO_3_ (67.6 M)/NaOH (5.0 M) (9/1 (v/v)) with a total volume of ca. 10 mL and the treatment was carried out at 90°C for 3 h. Subsequently, HCl solution (with a concentration equivalent to NaOH) was added into the above polymer solution. This was followed by dialysis (Cellu Sep MWCO 3500) against deionized water for 7 days. The poly(γ-GA) product was then collected by lyophilization. The molecular weight and polydispersity of the resultant poly(γ-GA) were determined by size exclusion chromatography (SEC) (Agilent 1100, PL Aquagel-OH columns in series GF083: separation range 100-30K; GF084: 10K-200K and GF086: 200K-10M, calibrated with poly(sodium acrylate) standards of known molecular weights with narrow molecular weight distributions (eluent: tris buffer 0.01 M, pH 7.4; flow rate: 1.0 mL/min; RI detector (Aglient 1100)).

### Synthesis of Lipid/Polypeptide Adduct

**Figure 1:**
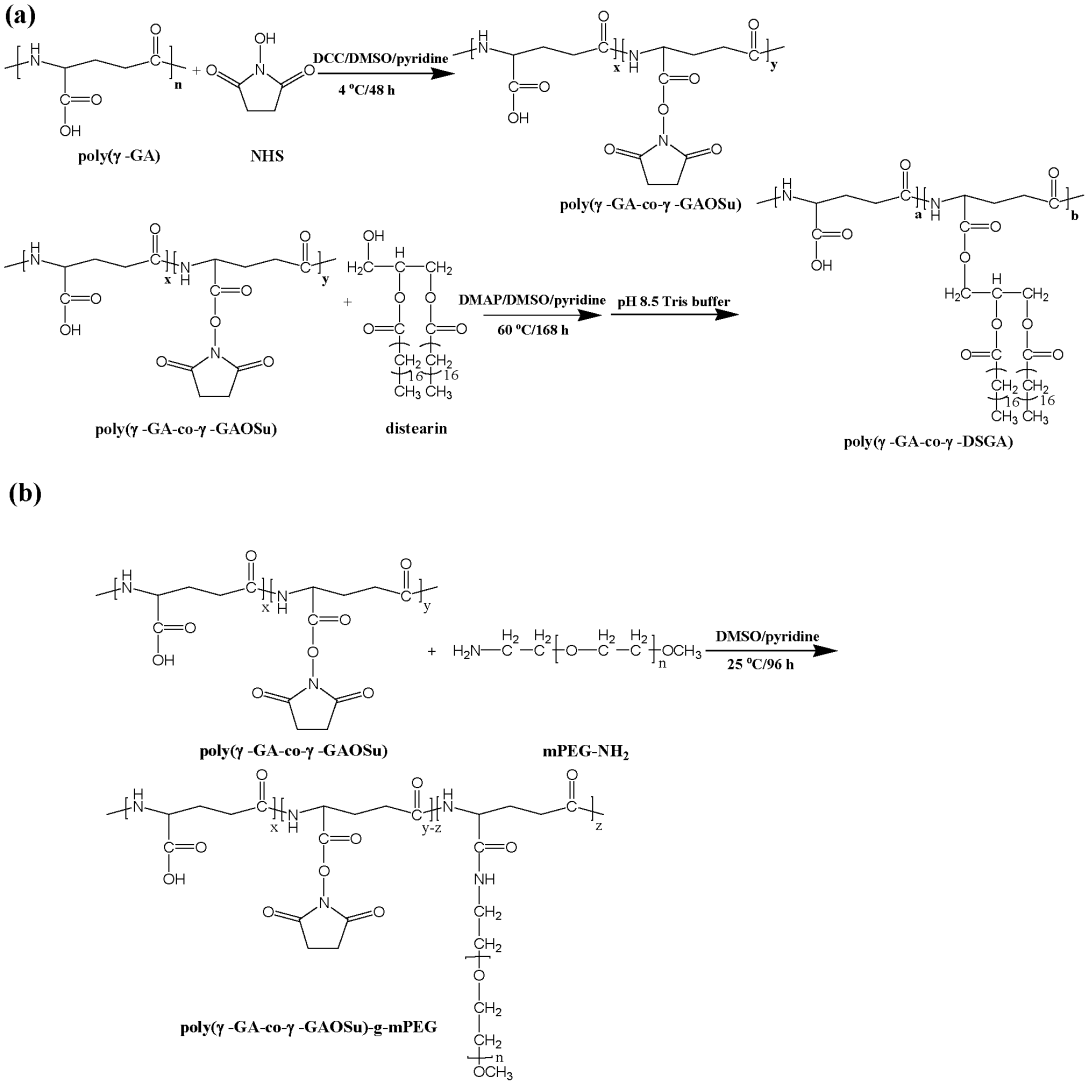
Synthetic route of (a) poly(γ-GA-co-γ-DSGA) and (b) poly(γ-GA-co-γ-GAOSu)-g-mPEG.

The synthetic route of poly(γ-GA-co-γ-DSGA) is illustrated in Figure 1a. To partially activate the γ-GA residues of poly(γ-GA) into the reactive γ-GAOSu moieties, NHS (50 mol % with respect to the γ-GA residues) and DCC (equal to molar concentration of NHS) used as the coupling agent were added to the solution of poly(γ-GA) in DMSO/pyridine (3/1 (v/v)). The reaction was carried out at 4°C for 48 h. The undesired product, *N,N’*-dicyclohexylurea (DCU), was then removed by the repeated filtration of the concentrated polymer solution. The partial transesterification of poly(γ-GA-co-γ-GAOSu) with distearin (25 mol% with respect to the original γ-GA residues in poly(γ-GA)) was then conducted at 60°C for 7 days, using 4-dimethylaminopyridine as the catalyst. This was followed by the dialysis (Cellu Sep MWCO 12000∼14000) against DMSO/THF (3/1 (v/v) solution) to eliminate the unreacted lipid species. Afterward, full hydrolysis of the remaining γ-GAOSu into the γ-GA residues was achieved by the addition of pH 7.4 tris buffer. The solution was then dialyzed (Cellu Sep MWCO 12000∼14000) against deionized water to remove NHS, DMSO and THF. Finally, the purified lipid/polypeptide conjugate was collected by lyophilization. The chemical composition was determined by ^1^H-NMR (Varian Unityinova 500 NMR Spectrometer) using DMSO-*d*_6_ as the solvent.

### Synthesis of Poly(γ-GA-co-γ-GAOSu)-g-mPEG

Coupling poly(γ-GA-co-γ-GAOSu) with mPEG-NH_2_ grafts (M_w_ 5000 g/mol) (10 mol% with respect to the original γ-GA residues in poly(γ-GA)) was conducted at 25°C for 96 h under stirring. The polymer solution was dialyzed (Cellu Sep MWCO 12000∼14000) against DMSO/ethanol (1/1 (v/v)) for 5 days to remove NHS and unreacted mPEG-NH_2_. The final product was collected by precipitation from ether/ethanol solution. The grafting reaction is illustrated in Figure 1b. The purity was confirmed by SEC. The chemical composition was determined by ^1^H-NMR using DMSO-*d*_6_ as the solvent at the ambient temperature.

### Preparation of DOX-Loaded Lipid/Polypeptide Vesicles

The lipid modified polypeptide (1.0 mg) was first dispersed in 1.0 mL phosphate buffer (pH 7.4, I 0.01 M) containing DOX (0.125 mg/mL) and then stirred for 12 h. The above dispersion was then subjected to ultrasonication with cooling in an ice-water bath for 5 min (VCX750Vibra-Cell Ultra Sonics Processor, a probe-type ultrasonicator) to produce the vesicle suspension. Afterward, the aqueous solution of the DOX-loaded vesicles was dialyzed (Cellu Sep MWCO 12000∼14000) against phosphate buffer (pH 7.4, I 0.01 M) for 12 h to remove unloaded DOX species. The DOX-loaded vesicles were dissolved completely by the addition of DMSO at a volume ratio of DMSO/H_2_O  =  9/1 and the amount of DOX was quantitatively determined by fluorescence measurements on a Hitachi F-7500 fluorescence spectrometer. The excitation was performed at 480 nm and the emission spectra were recorded in the range 500–700 nm. The calibration curve was established by standard DOX solutions of seven concentrations in the DMSO/H_2_O (9/1 (v/v)) solution. Drug loading efficiency (DLE) was calculated as follows: 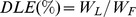



where W_L_ and W_F_ represent the weights of loaded DOX and DOX in feed, respectively.

### Preparation of DOX-Loaded GCPVs

Coating dual-layered nanogels on the DOX-loaded polymeric vesicles was achieved by depositing chitosan and poly(γ-GA-co-γ-GAOSu)-g-mPEG in sequence onto the vesicle surface via the complementary electrostatic attraction. The aqueous solution of chitosan (5.0 mg/mL, 4.0 mL, pH 5.0) was directly added into the above DOX-loaded vesicle suspension of pH 7.4 (6.0 mL) and the mixture was then stirred for 8 h. This was followed by the addition of the DOX-loaded chitosan-coated vesicle dispersion (1.0 mL) to the aqueous solution of poly(γ-GA-co-γ-GAOSu)-g-mPEG (3.5 mg/mL, pH 7.4). The resultant dispersion was then stirred at ambient temperature for 12 h to achieve the ionic polyelectrolyte complex formation and in situ covalent crosslinking via aminolysis of primary amines from chitosan with reactive γ-GAOSu residues. The aqueous dispersion of the DOX-loaded GCPVs was subjected to ultrafiltration (Amicon 8200 with a Millipore PBMK membrane, MCWO 300000) in phosphate buffer (pH 7.4, I 0.01 M) to eliminate the coating materials that were not cross-linked.

### Structural Characterization

The mean hydrodynamic diameter (D_h_), particle size distribution (PSD) and zeta potential of the pristine polymeric vesicles, the DOX-loaded vesicles and GCPVs in aqueous solutions were determined by a Malvern Zetasizer Nano-ZS instrument at a scattering angle of 173^o^ equipped with a 4 mW He-Ne laser operating at λ  =  632.8 nm. Prior to measurement, the sample was equilibrated at 25°C for 20 min. The data reported herein represent an average of at least triplicate measurements. To identify the morphology of polymeric assemblies, the ratio of the root-mean-square radius of gyration (R_g_) to hydrodynamic radius (R_h_) of particles was obtained by dynamic and static light scattering (DLS/SLS) measurements. Herein, R_h_ was determined by a Brookhaven BI-200SM goniometer equipped with a BI-9000 AT digital correlator using a solid-state laser (35 mW, λ  =  637 nm) at a scattering angle of 90°. The CONTIN algorithm method was employed for DLS data analysis in order to confirm the absence of bimodal particle size distribution of the polymeric assemblies with greatly enhanced reliability. As to R_g_, it was obtained from the angle-dependent measurement of the light scattering intensity. A Berry plot of the scattering intensity (I_ex_^1/2^) versus the square of scattering vector (q^2^) was used for quantitative determination of R_g_ [30]. In addition, the angular dependence of the autocorrelation functions was assessed using the same instrument as described above. Correlation functions were analyzed by the cumulant method at different angles.

### Transmission Electron Microscopy (TEM) Examination

The sample was prepared by placing a few drops of aqueous dispersion of the lipid/polypeptide conjugate assemblies on a 300-mesh copper grid covered with carbon and allowed to stand at 25°C for 20 s. Excess solution on the grid was gently removed with absorbent paper. This was followed by negative staining of the sample for 20 s using a uranyl acetate solution (2.0 wt %). The sample was then dried at 25°C for 2 days. The TEM image was acquired on a JEOL JEM-1200 CXII microscope operating at an accelerating voltage of 120 kV.

### In vitro Drug Release

The in vitro DOX release from the drug-loaded polymeric vesicles was assessed in buffer solutions. The phosphate buffer saline (PBS) of pH 7.4 (or the succinate buffer of 4.7) was used as the release medium in this study. The DOX-loaded polymeric vesicles (or GCPVs) dispersed in phosphate buffer (pH 7.4, 2.0 mL) was transferred to a cellulose membrane tube (Cellu Sep MWCO 12000–14000). The dialysis tube was then placed in the release medium (100 mL) and gently shaken (100 rpm) in a water bath at 37°C. At the preset time intervals, 1.0 mL of external buffer solution (either PBS or succinate buffer) was withdrawn and replaced with an equal volume of fresh medium. The concentration of DOX was determined by fluorescence measurements using the pertinent calibration curve of DOX with various concentrations in aqueous solution of either pH 7.4 or 4.7.

### Cellular Uptake Study

HeLa cells (1.0×10^5^ cells/well) were incubated in culture medium for 24 h and then treated with free DOX and the DOX-loaded vesicles and GCPVs, respectively, at a DOX concentration of 10 μM at 37°C for either 1 or 2 h. After being washed twice with PBS, cells were detached by trypsin-EDTA solution and dispersed in 0.5 mL of PBS. The cellular uptake of DOX was examined by a FACSCalibur flow cytometer (BD Bioscience). For confocal laser scanning microscopy (CLSM) studies, HeLa cells (1×10^5^ cells/mL) were cultured in DMEM in glass bottom culture dishes at 37 °C for 24 h. The medium was then removed and the cells were washed twice with PBS. Cells were treated with free DOX and the DOX-loaded GCPVs, respectively, at a DOX concentration of 10 μM at 37°C for 2 h. Cells were washed with PBS, fixed with 4% formaldehyde and then stained with Hoechst 33258 for 10 min. The cellular uptake of DOX was visualized by OLYMPUS FV1000 confocal imaging system (Olympus, Japan) at the excitation and emission wavelength of 488 and 590 nm, respectively.

### In vitro Cytotoxicity Evaluation

The in vitro cytotoxicity of the DOX-loaded GCPVs and DOX-free GCPVs was evaluated by MTT assay with HeLa cells. For comparison, free DOX was used as a positive control. HeLa cells were seeded in 96-well plates (1×10^4^ cells/well) and incubated at 37°C for 24 h. The medium was then replaced with 100 μL of fresh medium containing free DOX or the DOX-loaded GCPVs at an equivalent DOX concentration ranging from 0.01 to 10 μM or the DOX-free GCPVs, and cells were incubated for additional 48 h. Afterward, 10 μL of MTT (5.0 mg/mL) was added into each well, followed by incubation at 37°C for 4 h. Following the removal of the supernatant, 100 μL of DMSO was added into each well to dissolve the precipitate. The optical density (OD) at 570 nm was measured with a SpectraMax M5 microplate reader.

## Results and Discussion

### Synthesis and Characterization of Poly(γ-GA-co-γ-DSGA) and Poly(γ-GA-co-γ-GAOSu)-g-mPEG

**Figure 2:**
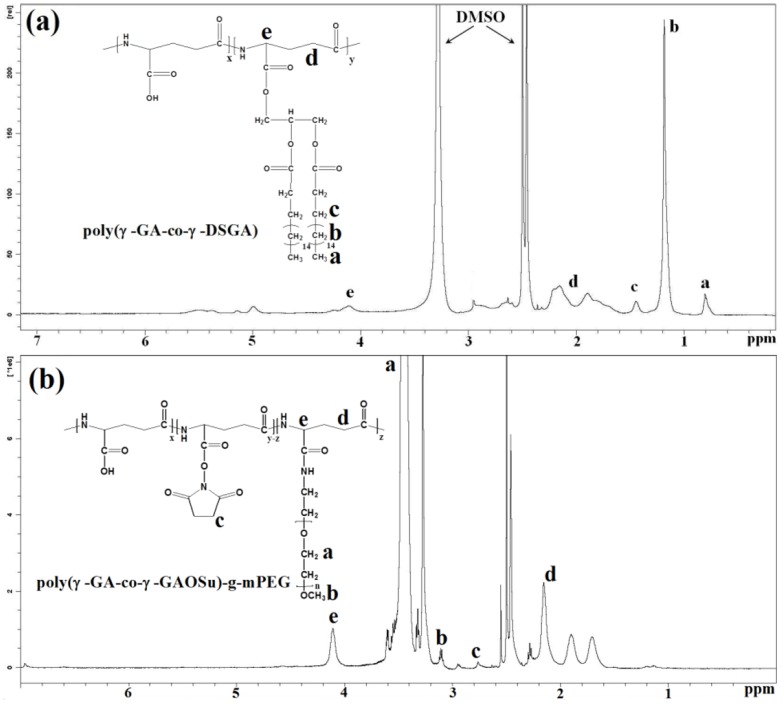
^1^H-NMR spectra of (a) poly(γ-GA-co-γ-DSGA) and (b) poly(γ-GA-co-γ-GAOSu)-g-mPEG in DMSO-*d_6_* at 25°C.

The relative molecular weight and polydispersity of the fractionated poly(γ-GA) were determined by SEC to be ca. 15600 g/mol and 1.46, respectively. Through partial transesterification of poly(γ-GA-co-γ-GAOSu) with distearin and subsequent full hydrolysis of the residual γ-GAOSu moieties into γ-GA residues in tris buffer of pH 7.4, the lipid/polypeptide conjugate, poly(γ-GA-co-γ-DSGA), was successfully attained. The ^1^H-NMR spectrum of this distearin-grafted polypeptide in DMSO-d_6_ is shown in Figure 2a. Based on the integral ratio of the feature signals of glutamic α proton of γ-GA at δ 4.1 ppm and methyl protons of distearin grafts at δ 0.81 ppm, the lipid content was estimated to be ca. 18.0 mol% with respect to the initial γ-GA residues. On the other hand, the mPEG-grafted polypeptide, poly(γ-GA-co-γ-GAOSu)-g-mPEG, was synthesized by the partial aminolysis of reactive γ-GAOSu moieties (24.0 mol% obtained from ^1^H-NMR analysis) of poly(γ-GA-co-γ-GAOSu) with mPEG-NH_2_. Figure 2b shows the ^1^H-NMR spectrum of poly(γ-GA-co-γ-GAOSu)-g-mPEG in DMSO-d_6_. Based on the signal integral ratio of glutamic α proton of γ-GA at δ 4.1 ppm to ethylene protons of mPEG at δ 3.5 ppm, the mPEG content amounting to ca. 4.0 mol% in the graft copolymer was attained. The recipes, compositions and average molecular weights of these two derived polypeptide adducts are summarized in Table 1.

**Table 1:** Recipes, compositions and average molecular weights of the derived polypeptide adducts.

sample	reaction feed (mol %)	composition ratio^a^ (mol %)	M_w_ (×10^−4^ g/mol)
poly(γ-GA-co-γ-DSGA)	γ-GA/NHS/distearin	γ-GA/DSGA	
	100/50/25	82/18	2.81^b^
poly(γ-GA-co-γ-GAOSu)-g-mPEG	γ-GA/NHS/mPEG	γ-GA/γ-GAOSu/mPEG	
	100/30/10	76/20/4	4.09^c^

a Determined by ^1^H-NMR in DMSO-*d_6_* at 20°C.

b Obtained by theoretical calculation as follows: poly(γ-GA) M_w_ 15600 (g/mol) + number of conjugated distearin moieties × 625 (M_w_ of distearin)

c Obtained by theoretical calculation as follows: poly(γ-GA) M_w_ 15600 (g/mol) + number of conjugated Osu moieties ×114 (M_w_ of OSu) + number of mPEG grafts ×5000 (M_w_ of mPEG).

### Preparation and Characterization of DOX-Loaded Polymeric Vesicles

**Figure 3:**
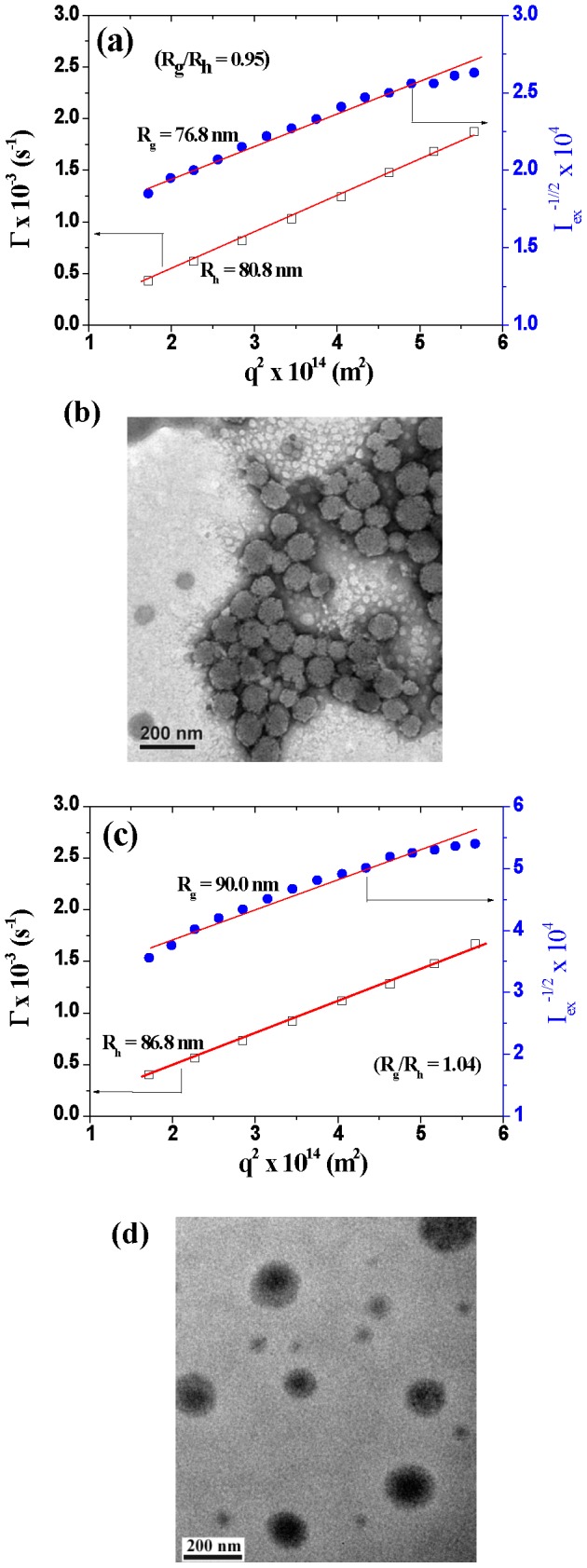
Angle-dependent DLS/SLS data and TEM images of lipid/polypeptide conjugate vesicles (a, b) and DOX-loaded GCPVs (c, d).

By virtue of the inherent hydrophobic nature of distearin covalently conjugated to a significant extent with the hydrophilic polypeptide segments, it is anticipated that such a lipid/polypeptide adduct exhibits strong amphiphilic characteristic in aqueous phase. This distearin grafted polypeptide (1.0 mg/mL) subjected to the probe-type sonication in phosphate buffer of pH 7.4 underwent hydrophobic association of γ-DSGA residues into supramolecular packing assemblies with particle size of ca. 150 nm in D_h_ and PSD of ca. 0.24 (Table 2). Notably, a linear relationship between the relaxation frequency (Γ) and the square of the scattering vector (q^2^) in the angle-dependent DLS data (Figure 3a) demonstrates the assembly morphology in a spherical particle form [10], [15], [30]. This was further confirmed by the TEM examination, as shown in Figure 3b. In addition, the mean particle size of lipid/polypeptide conjugate assemblies examined by TEM is slightly smaller than those measured by DLS. This discrepancy is because of the transition of assemblies from the swollen (DLS) to dried state (TEM). In order to gain a fundamental insight into the molecular packing architecture of colloidal assemblies in aqueous solution (pH 7.4), their R_g_ and R_h_ were evaluated by SLS and DLS measurements, respectively. R_g_ is intimately associated with the mean spatial distance from the mass center of the target assembly to individual atoms (groups) that constitute the assembly, whereas R_h_ represents the radius of a hypothetical hard sphere that diffuses with the same speed as the target under examination. It is therefore readily understood that the R_g_/R_h_ ratio is susceptible to the assembly topology and this parameter can be employed for the structure assessment [10], [15], [31], [32]. As revealed in Figure 3a, the R_g_/R_h_ ratio (ca. 0.95) of the lipid/polypeptide conjugate assemblies in aqueous solution that is quite close to the theoretical value (1.0) for thin-layer hollow spheres [31], [32] distinctly demonstrates the structure in a vesicle-like morphology. Compared to the γ-GA residues, the γ-DSGA moieties are obviously more hydrophobic, thus being prone to self-assemble into the non-polar internal membrane layer structure during the molecular packing process of the lipid-derived polypeptide adduct. It was reported that polymer vesicles attained from amphiphilic block copolymers were generally characterized in membrane morphology by continuous hydrophobic internal layers surrounded by hydrophilic coronae, while graft copolymers very often undergo the vesicle formation with the membrane structure comprising discrete nonpolar islets embedded within the hydrophilic matrix [13],[33]. This difference is primarily due to the configurational dissimilarity between these two types of amphiphilic copolymers. In spite of more resembling the latter, the objective of this work was not to study the detailed vesicle structure, but to develop functional drug-loaded nanovehicles for effective intracellular payload release instead. Nevertheless, a relatively high negative zeta potential of the polymeric hollow colloids at pH 7.4 was detected (Table 2). This implies that the vesicle surfaces are abundant in the negatively charged γ-GA residues that can serve as facile binding sites for the oppositely charged anticancer agents.

**Table 2:** D_h_, PSD and zeta potential of the polymeric vesicles, DOX-loaded vesicles, DOX-loaded chitosan-caged vesicles and DOX-loaded GCPVs in aqueous solutions at 25°C.

Sample	D_h_ (nm)^a^	PSD	Zeta potential (mV)
Pristine vesicles at pH 7.4	150±7	0.24	–24±4
DOX-loaded vesicles at pH 7.4	142±4	0.25	–18±4
DOX-loaded chitosan-caged vesicles at pH 6.0	149±5	0.20	+26±3
DOX-loaded GCPVs at pH 7.4	168±5	0.17	–5±1

a Determined by DLS (Malvern Zetasizer Nano-ZS instrument) at a scattering angle of 173^o^, using the cumulant analysis method.

To encapsulate DOX molecules into the biomacromolecular conjugate vesicles, the phosphate buffer containing DOX (0.125 mg/mL, pH 7.4) was employed instead of the drug-free phosphate buffer used in the procedure for preparing polymeric vesicles described above. As shown in Table 2, both particle size and particle size distribution of the drug-loaded vesicles are comparable to those of the pristine counterpart. This indicates that encapsulation of DOX does not significantly interfere the development of polymeric vesicles under the conditions adopted in this study. The polymeric vesicles exhibit an appreciable reduction in the zeta potential after drug loading. This indicates that the positively charged DOX species are not only confined within the inner aqueous chamber of polymeric vesicles but also partly bound with the ionized γ-GA residues via the cooperative electrostatic attraction [34], [35]. Hence, such a high drug loading efficiency amounting to ca. 80% corresponding to a payload quantity of 10.0 wt % was achieved by the approach proposed in this work.

### In vitro Drug Release of DOX-Loaded Polymeric Vesicles

**Figure 4:**
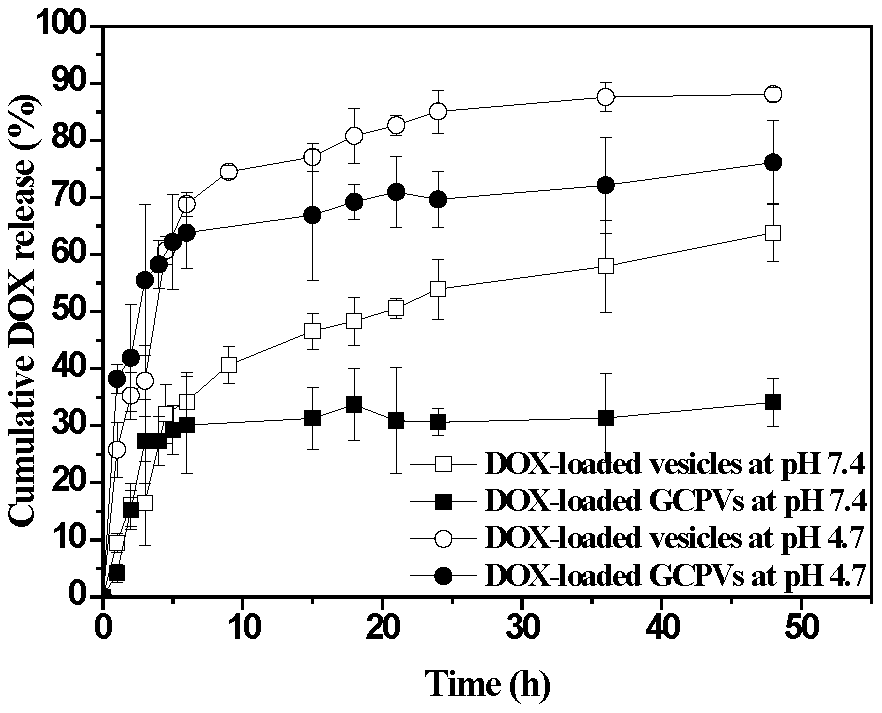
Cumulative drug release profiles of DOX-loaded vesicles and DOX-loaded GCPVs in aqueous solutions of pH 7.4 and 4.7, respectively, at 37°C.

To assess the drug release performance, the aqueous suspension of the DOX-loaded assemblies placed in the dialysis tubes was immersed into the buffer solution of pH 7.4 or 4.7 (ionic strength of 0.15 M) at 37°C. As shown in Figure 4, the DOX-loaded vesicles at pH 4.7 over a period of 10 h exhibit a significantly higher cumulative drug release (ca. 75%) than those at pH 7.4 (40%). This is because, in addition to the increased aqueous solubility of DOX with the medium pH being reduced from 7.4 to 4.7, an appreciable reduction in the ionization extent of γ-GA residues leads to the massive disruption of the original electrostatic interaction between γ-GA residues and DOX species and, thus, accelerates drug liberation. Although the DOX-loaded polymeric vesicles show a prominent pH-responsive drug release behavior, more than 60% of the payload is released at pH 7.4 within 48 h. Such a substantial level of drug liberation at pH 7.4 implies severe impairment of the lipid/polypeptide conjugate vesicles in drug delivery application. This is attributed to the premature drug leakage in the blood stream prior to arrival at the target tumors, which obviously may cause undesired side effect and reduce therapeutic efficacy.

### Development of DOX-Loaded GCPVs and Their in vitro Drug Release

In order to overcome the aforementioned drawback, the DOX-loaded lipid/polypeptide vesicles were further functionalized with the pH-responsive dual-layered gels acting as a spatial valve-like controller with capability to retard drug release at pH 7.4 (e.g. in blood circulation system), but allow cargo release in weak acidic media (e.g. intracellular endosomal/lysosomal compartments). To achieve this goal, chitosan and poly(γ-GA-co-γ-GAOSu)-g-mPEG were sequentially layered onto the outer surfaces of the drug-loaded vesicles to form polyelectrolyte complexes via the cooperative electrostatic interaction at pH 7.4. The in situ covalent cross-linking was simultaneously achieved upon the aminolysis of γ-GAOSu moieties with primary amines from chitosan. The step-by-step polyelectrolyte deposition on the outer surfaces of polymeric vesicle was monitored by DLS and zeta potential measurements. As shown in Table 2, incorporation of the outlayered polyelectrolyte gels increases the particle size from 142 to 168 nm in D_h_. The zeta potential of the DOX-loaded polymeric vesicles with the intermediate deposition of chitosan is shifted from ca. –19 to +26 mV. Furthermore, after being combined with poly(γ-GA-co-γ-GAOSu)-g-mPEG, the zeta potential of the chitosan-coated DOX-loaded polymeric vesicles then changes from +26 to ca. –5 mV. Similar results with the development of the layer-by-layer structure on nanoparticle surfaces via the electrostatic interaction that gave rise to the increase of particle size and the inversion of nanoparticle surface charges were also reported elsewhere [36], [37]. Herein, it should be mentioned that the spatial barrier of intermediate chitosan-based layers and the stable pairing of DOX with γ-GA residues of vesicles at pH 7.4 could efficiently inhibit the covalent conjugation of γ-GAOSu moieties with loaded DOX during the polyelectrolyte deposition process. The linear relationship of the Γ versus q^2^ data (Figure 3c) and the TEM image (Figure 3d) of the drug-loaded GCPVs clearly demonstrate that the resultant complex assemblies still possess a spherical shape after being decorated with the dual-layered gels. Moreover, the TEM imaging contrast of drug-loaded GCPVs was significantly amplified compared to that of polymeric vesicles. In addition to the staining difference, it is also attributed to that the encapsulated DOX molecules in the interior of the drug-loaded GCPVs increase the electron scattering level of nanoparticles, thus promoting TEM imaging contrast. Note that the R_g_/R_h_ value (1.04) of the DOX-loaded GCPVs in aqueous solution of pH 7.4 (Figure 3c) illustrates that the polymer complexes with supplemented layered gels retain the original hollow sphere architecture. Importantly, the DOX-free and DOX-loaded GCPVs after being subjected to large-volume dilution with FBS-containing (10%) PBS at 37°C still retain unchanged in particle size for at least ten days. This strongly indicates that the amide linkage-containing outlayered gels and the outmost hydrophilic PEG chain segments endow the GCPVs with better colloidal stability and serum protein-repellent property. In this regard, it is expected that the DOX-loaded GCPVs have a prolonged blood circulation time, enabling to promote the therapeutic efficacy of cancer treatment by enhanced permeability and retention effect.

**Figure 5:**
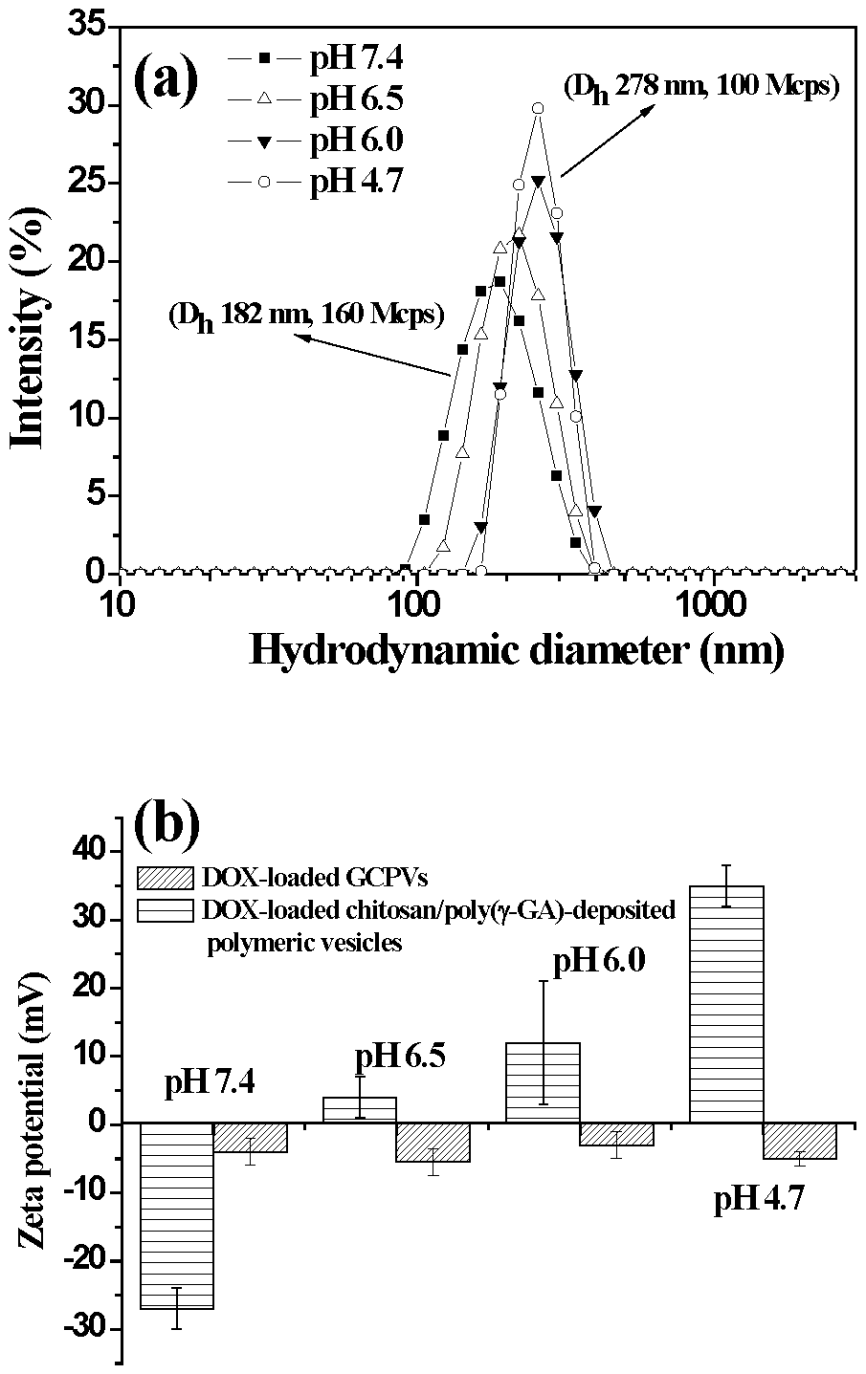
pH-dependent characteristics of DOX-loaded GCPVs and DOX-loaded chitosan/ poly(γ-GA)-deposited polymeric vesicles. (a) DLS colloidal particle size distribution profiles of DOX-loaded GCPVs in aqueous media of various pH. (b) Zeta potentials of DOX-loaded GCPVs and DOX-loaded chitosan/ poly(γ-GA)-deposited polymeric vesicles in different pH aqueous media.

**Figure 6:**
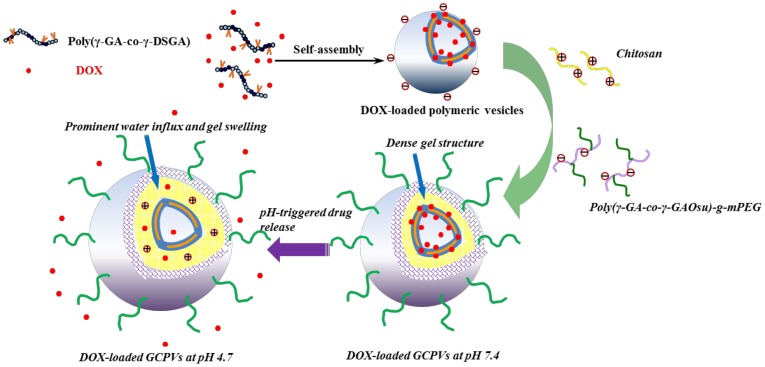
Schematic illustration of the development of DOX-loaded GCPVs and their pH-triggered drug release.

Figure 4 shows the effect of the dual-layered gels of the DOX-loaded GCPVs on the in vitro pH-involved drug liberation. The cumulative drug release (ca. 28%) of the DOX-loaded GCPVs at pH 7.4 over a period of 48 h is much lower than that (ca. 60%) of the DOX-loaded vesicles under exactly the same conditions. Furthermore, at pH 4.7, the DOX-loaded GCPVs exhibit a prompt drug release profile comparable to the DOX-loaded polymeric vesicles prior to the nanogel coating. These results manifest that the dual-layered gels indeed play a vital role in fine tuning the drug release in response to the external pH change. The γ-GA/DOX ionic complexes remained to some extent within either polymeric vesicles or GCPVs at pH 4.7, thus giving rise to an incomplete unloading of DOX species from these nanovehicles. Figure 5a shows that, with the external pH being decreased from pH 7.4 to 4.7 (ionic strength 0.15 M), the particle size of the DOX-loaded GCPVs is remarkably enlarged from 182 to 278 nm along with the light scattering intensity being appreciably reduced from 160 to 100 Mcps. By contrast, only slight variations in the particle size and light scattering intensity of the DOX-loaded vesicles without the outlayered gels were observed when the pH decreased from pH 7.4 to 4.7 (data not shown here). This result clearly confirms the swelling of the outlayered gels of GCPVs in weak acidic environment. With the medium pH being switched from 7.4 to 4.7, the massive disruption of the chitosan/poly(γ-GA) complexes and the ionic osmotic pressure gradient developed within the interfacial polysaccharide layers occur primarily due to the increased protonation of both the γ-GA residues and the primary amino groups from chitosan chain segments, thereby rendering the outlayered gels themselves more hydrated and swollen. Furthermore, the zeta potential of the DOX-loaded GCPVs in response to the pH change remained in the range 0∼–10 mV (Figure 5b). This signifies that despite the significant disruption of the polyelectrolyte complexes, the outlayered gel structure still can be preserved without dissociation into individual macromolecular unimers, even at low pH. Under the circumstance, the charge-shielding effect of the outmost mPEG chain segments is maintained. By contrast, in the absence of both mPEG chain segments and covalent crosslinking between the two external layers, the chitosan/poly(γ-GA)-deposited polymeric vesicles became unstable with the pH being adjusted from 7.4 to 4.7 as evidenced by showing the dramatic change in zeta potential from ca –27 (pH 7.4) to 35 mV (4.7) owing to large disruption of the non-crosslinked chitosan/poly(γ-GA) complexes and detachment of the poly(γ-GA) layer from the particle surfaces (Figure 5b). Based on the above results, we have demonstrated that coating these robust pH-responsive dual-layered gels onto the DOX-loaded polymeric vesicles not only prevents the payload from premature leakage at pH 7.4 by their effectively protective dense structure but also retains the passage for drug release at pH 4.7 by the pertinent gel swelling (Figure 6).

### Cellular Uptake

**Figure 7:**
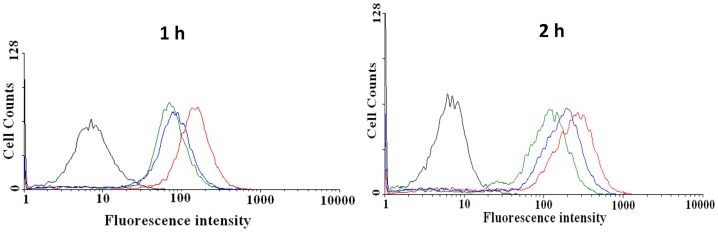
Flow cytometric histogram profiles of HeLa cells incubated with free DOX, DOX-loaded vesicles and DOX-loaded GCPV. DOX fluorescence intensity of HeLa cells incubated with free DOX (red), DOX-loaded vesicles (green) and DOX-loaded GCPV (blue) at 37°C for 1 and 2 h, respectively. Untreated cells (black) were used as a control.

**Figure 8:**
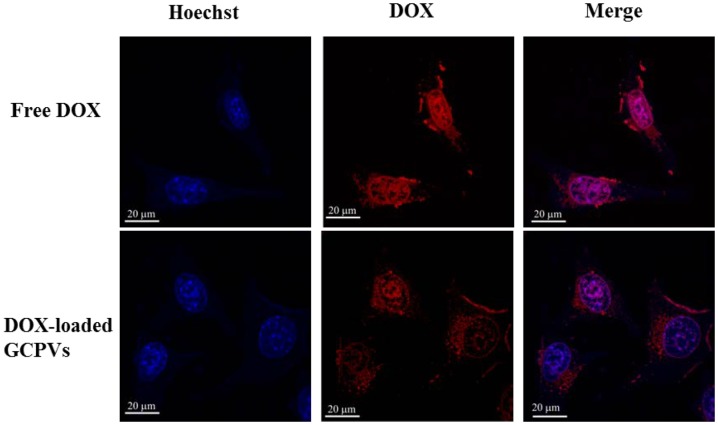
CLSM images of HeLa cells incubated with free DOX and DOX-loaded GCPVs at 37°C for 2 h (DOX concentration  =  10 μM). Cell nuclei were stained with Hoechst 33258.

To gain a better insight into the cellular uptake of the DOX-loaded GCPVs, the flow cytometry and CLSM measurements were conducted, using HeLa cells as a cancer cell model. Figure 7 shows the flow cytometric histograms of the DOX fluorescence of HeLa cells incubated with free DOX and the DOX-loaded vesicles and GCPVs at 37°C for 1 and 2 h, respectively. Cells alone without any DOX treatment were used as a negative control. With the same incubation time, appreciably higher cellular uptake of free DOX compared to the DOX-loaded vesicles and GCPVs is ascribed to two distinct internalization pathways. Free DOX is well known to be transported into cells via passive diffusion [10], [38], whereas the DOX-loaded vesicles and GCPVs are predominantly internalized by cells through an energy-dependent endocytic process. Moreover, with the incubation time being prolonged from 1 to 2 h, the cellular uptake of the DOX-loaded GCPVs was appreciably enhanced compared to that of the DOX-loaded vesicles. This could be ascribed to the different surface features of nanoparticles. For the DOX-loaded polymeric vesicles, the ionized γ-GA-rich surfaces partially reduce the cellular uptake by the electrostatic repulsion against the negatively charged membranes of cells [39]. By contrast, the DOX-loaded GCPVs exhibit a relatively neutral surfaces that certainly diminish the electrostatic repulsion with cell membranes, in addition to the inherent amphiphilic nature of the attached PEG coronae enhancing the particle surface contact with cell membrane [40]. On the other hand, the CLSM images (Figure 8) illustrate that the DOX fluorescence intensity within HeLa cells treated with drug-loaded GCPVs (for a time period of 2 h) was somewhat lower than that of cells incubated with free DOX. In addition, being distinct from the profound accumulation of free DOX molecules within the nuclei of HeLa cells by passive diffusion, the appreciable depositions of the drug species transported by GCPVs in both cytoplasm and nucleus regions were observed as a result of the acid-triggered drug release of the endocytosed GCPVs within acidic endosomes and lysosomes. These results are in well agreement with the flow cytometry measurements of intracellular DOX fluorescence intensity as shown in Figure 7.

### In vitro Cytotoxicity

**Figure 9:**
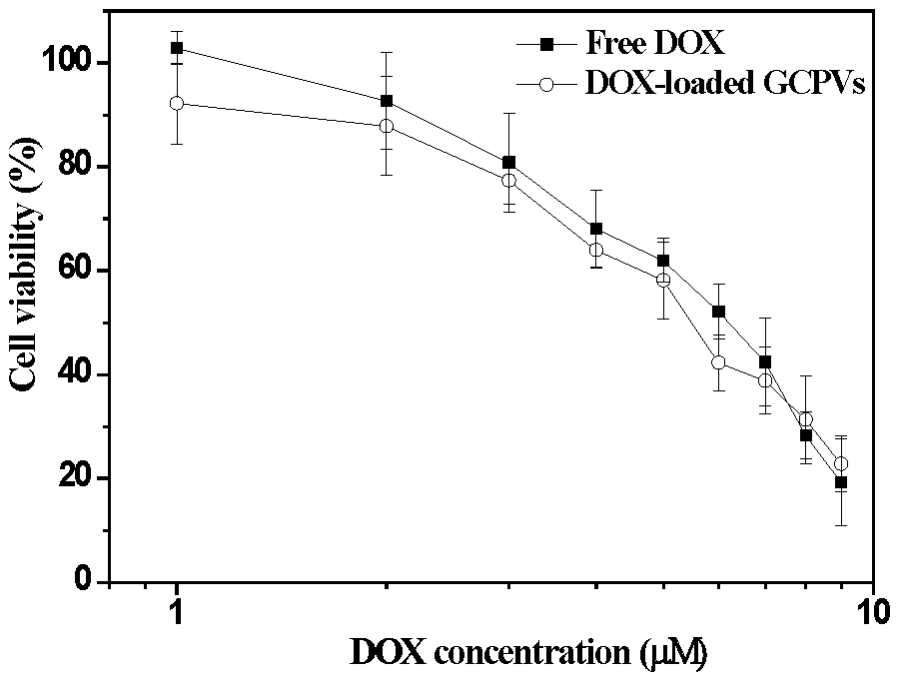
In vitro cytotoxicity of free DOX and DOX-loaded GCPVs against HeLa cells with an incubation time of 48 h. The data presented herein represent an average of at least triplicate experiments.

The cytotoxicity of free DOX and the DOX-loaded GCPVs against HeLa cells evaluated by MTT assay is presented in Figure 9. As a crucial control, the high viability of HeLa cells incubated with drug-free GCPVs even at high concentrations was observed (data not shown here). This implies that the pristine GCPVs be virtually nontoxic to HeLa cells. The drug doses for 50% cellular proliferation inhibition (IC_50_) of free DOX and the DOX-loaded GCPVs were observed to be ca. 0.63 and 0.45 μM, respectively. In comparison with other DOX vehicles that exhibited lower cytotoxicity than free DOX due to slow drug liberation from carriers and delayed nuclear uptake in HeLa cells [41], [42], the DOX-loaded GCPVs showed the capability of efficiently inhibiting cell proliferation comparable to free DOX. Based on the results of the in vitro drug release (Figure 4) and cellular uptake (Figure 7), it is expected that a high intracellular drug concentration can be attained by virtue of the pH-triggered prompt DOX release from GCPVs while residing within intracellular acidic endosomes and lysosomes, thereby maximizing the therapeutic efficiency [43].

## Conclusion

In this study, the drug-loaded lipid/polypeptide conjugate vesicles were further augmented with the pH-responsive dual-layered gels for highly efficient intracellular drug delivery. Through the sequential deposition of chitosan and poly(γ-GA-co-γ-GAOSu)-g-mPEG in combination with in situ covalent cross-linking on the outer surfaces of the DOX-loaded vesicles, the drug-loaded GCPVs were successfully attained. The GCPVs exhibited superior capability of controlling drug release in response to the external pH change. The additional pH-responsive dual-layered gels not only effectively prevent the drug payload from premature leakage at pH 7.4 by the dense gel structure but also retain facile passage of DOX release at pH 4.7 due to the pertinent gel swelling. The GCPVs after being internalized by HeLa cells via endocytosis showed prominent antitumor ability comparable to free DOX by rapidly releasing payload in intracellular acidic organelles. This work demonstrates the great potential of GCPVs as an intracellular drug delivery nanovehicle for improved anticancer treatment.
